# Organic farming practices change the soil bacteria community, improving soil quality and maize crop yields

**DOI:** 10.7717/peerj.11985

**Published:** 2021-09-23

**Authors:** Ademir Durrer, Thiago Gumiere, Maurício Rumenos Guidetti Zagatto, Henrique Petry Feiler, Antonio Marcos Miranda Silva, Rodrigo Henriques Longaresi, Sérgio K. Homma, Elke J.B.N. Cardoso

**Affiliations:** 1 Soil Science Department/ Luiz de Queiroz College of Agriculture, University of São Paulo, Piracicaba, São Paulo, Brazil; 2 Department of Soil and Agricultural Engineering, Laval University, Quebec City, Canada, Canada; 3 Mokiti Okada Research Center, Ipeúna, São Paulo, Brazil

**Keywords:** Bacterial community, Bokashi, Composted poultry manure, Green manure, Nature farming, Soil enzymes, Soil management systems

## Abstract

**Background:**

The importance of organic farming has increased through the years to promote food security allied with minimal harm to the ecosystem. Besides the environmental benefits, a recurring problem associated with organic management is the unsatisfactory yield. A possible solution may rely on the soil microbiome, which presents a crucial role in the soil system. Here, we aimed to evaluate the soil bacterial community structure and composition under organic and conventional farming, considering the tropical climate and tropical soil.

**Methodology:**

Our organic management treatments were composed by composted poultry manure and green manure with Bokashi. Both organic treatments were based on low nitrogen inputs. We evaluated the soil bacterial community composition by high-throughput sequencing of 16S rRNA genes, soil fertility, and soil enzyme activity in two organic farming systems, one conventional and the last transitional from conventional to organic.

**Results:**

We observed that both organic systems evaluated in this study, have higher yield than the conventional treatment, even in a year with drought conditions. These yield results are highly correlated with changes in soil chemical properties and enzymatic activity. The attributes pH, Ca, P, alkaline phosphatase, and β- glucosidase activity are positively correlated with organic systems, while K and Al are correlated with conventional treatment. Also, our results show in the organic systems the changes in the soil bacteria community, being phyla *Acidobacteria, Firmicutes, Nitrospirae,* and* Rokubacteria* the most abundant. These phyla were correlated with soil biochemical changes in the organic systems, helping to increase crop yields.

**Conclusion:**

Different organic management systems, (the so-called natural and organic management systems, which use distinct organic sources), shift the soil bacterial community composition, implying changes in their functionalities. Also, our results contributed to the identification of target bacterial groups and changes in soil chemical properties and enzymatic activity in a trophic organic farming system, which may contribute to higher crop yields.

## Introduction

Agriculture is crucial for human life, contributing to food and energy production, as well as the obtention of many manufactured products. After the green revolution, agricultural productivity is increasing due to high applications of synthetic fertilizers and pesticides, improvements in irrigation, and new soil management practices (Tilman et al., 2002). With this agricultural intensification, new concerns about the impacts on soil ecosystem services, beneficial effects for agriculture due to biological soil factors and living organisms in the environment, as well as loss of productivity after a long period of cultivation are appearing (Pervaiz et al., 2020). Intensive agricultural practices generally impact soil ecosystem services, including soil degradation, biodiversity loss, poor nutrient cycling, greenhouse gas emissions, pesticide accumulation, and nutrient leaching (Davis & Fox, 2009). Intensive tillage and chemical fertilization often lead to surface runoff and nutrient leaching especially in tropical conditions (Ramírez-Ávila & Sotomayor-Ramírez, 2011). It has been estimated that one-third of the eroded soil and chemicals are discharged into rivers and lakes, thus also impacting the aquatic environment (Kok, Papendick & Saxton, 2009).

As an alternative, some practices have been adopted to improve soil ecosystem services, including organic farming and reduced or no-tillage (Hobbs, Sayre & Gupta, 2008; Reganold & Wachter, 2016). These practices aim to enhance soil fertility and stimulate soil biology, maintaining high crop yields (Fließbach et al., 2007; Pan, Smith & Pan, 2009). Organic farming focuses on the natural equilibrium of the soil-plant system, producing high-quality and healthy food by banning harmful residues and toxic substances for humans and animals (Mondelaers, Aertsens & Van Huylenbroeck, 2009). In pratice, organic farming adopt the substitution of synthetic chemical fertilizers and pesticedes by fertilization with organic matter and biological controls, as well as, avoid genetically modified organisms.. Concurrently, soil fertility for the organic systems is sustained through organic matter addition, crop rotation, and biological disease control. It has been estimated that about 6.98 × 10^7^ ha of farmlands are managed under organic systems worldwide, with constant increases in these numbers (Willer, Yussefi-Menzler & Sorensen 2008). This tendency is a demand by the consumers for healthy food and scientific aspects showing environmental preservation, such as an improvement in soil quality (Doran & Parkin, 1994; Cardoso et al., 2013).

Distinct sources of organic matter are adopted as fertilizers to substitute the mineral fertilization and to establish an organic system. Composted animal manure (*e.g.,* poultry, pig or cow) and green manure or mixes with straw are the most recommended fertilizers to be used in organic practices (Govasmark et al., 2011; Zhen et al., 2014; Sacco et al., 2015; Chen et al., 2020). Organic fertilizers should have a C/N ratio of fewer than 30 parts of carbon for each part of nitrogen by weight, mainly when we aim to increase mineralization processes. However, other factors, such as enzyme activities, should be considered (Pfeiffer et al., 2013; Hartmann et al., 2015), since soil microorganisms play a crucial role in sustaining favorable soil conditions (*e.g.*, soil aggregation, biological control, nutrient cycling, organic matter decomposition, nitrogen fixation) (Bender, Wagg & Van der Heijden, 2016). In this view, the opportunity to use soil management practices that improve the microbial community and its functions can be a promissory approach for better crop yield (Panke-Buisse et al., 2015). If soil is rich in organic matter and all biological and biochemical processes are working well, we consider that the soil is healthy, containing an efficient microbiome and presenting all ecosystem services for maximum productivity (Tuomisto et al., 2012). Thus, healthy soils also maintain a harmonic functioning of microorganisms, enzyme activity, and soil fertility. More recently, comparisons between organic and conventional farming, using new technics of next-generation sequencing (NGS) have demonstrated changes in microbial community structure (Vega-Avila et al., 2015; Fernandez et al., 2016; Lupatini et al., 2017). Organic farming practices have demonstrated beneficial effects by enhancing soil organic matter content, soil porosity, structural stability, moisture, nutrient availability, biological activity, and soil erosion reduction (Reganold, Elliott & Unger, 1987; Marinari et al., 2006; Fließbach et al., 2007; Wang et al., 2017). Despite the benefits, organic farming has been known as presenting low productivity of crops. It has been observed that conventional farming yields generally are about 20% higher than those of organic farming (Mondelaers, Aertsens & Van Huylenbroeck, 2009; Tuomisto et al., 2012). In conventional management, each crop has a personalized prescription for the amount of each nutrient input and the correct timing for each farm activity. Meanwhile, for organic systems, each farmer uses a different kind of organic material, without considering the type, quality, and quantity, which disregards many aspects of soil organic matter cycling to supply plant growth. Thus, a study focused on the quality and quantity of the organic matter associated with the soil microbial community may reveal the reason for low productivity, and help to guide the management of organic systems.

Our study included two organic systems called 1. natural farming—fertilized with green manure and bokashi, and 2. organic—fertilized with a composted poultry manure. In opposition to these, we included the conventional cropping system with mineral synthetic fertilization representing the usual technique of maize production (one of the most important agronomic crops in the world). Finally, we analyzed the effect of the transition from conventional to organic farming, which consists of a five-year transitional period, slowly transforming the management from conventional to organic with composted poultry manure. At the beginning of our study, we were starting the second year of this system.

We evaluated changes in the bacterial community, soil quality indicators, and crop yields in all four management systems. We hypothesized that organic farming (1) promotes a productivity equivalent or superior to conventional farming, at the same time maintaining the soil health, as well as, (2) the soil fertility and soil biology are improved during the transition from conventional to organic farming. We also hypothesized that (3) different organic management systems (the so-called natural and organic management systems), using distinct organic sources, change the soil bacterial community, which implies changes in their functionalities.

## Materials and Methods

### Site description and experimental design

The study was conducted at Mokiti Okada Research Center located in Ipeúna city (SP) Brazil (22°24′5.42″S/ 47°40′51.65″W). According to the International Soil Taxonomy, the soils of the experimental fields are classified as Oxisols (Soil Survey Staff, 2010), presenting a clay loam texture. The experiment consisted of four treatments, each with 55,000 plants/ha with a distance of 0.7 m between lines. The maize cultivar used was the non-transgenic hybrid Agricon340. All treatments were set up during the crop-year 2017–2018 and 2018–2019, occupying 6 hectares. It is important to highlight that the occupied site by each experimental treatment measured 1.5 ha, *i.e.,* the size of a commercial maize plantation. This was only possible because the supervision of the experiment was done by the Research Center Mokiti Okada, our partner in this study . In this active crop region, 12 rectangular experimental plots were stablished (6 m × 7 m) considering three replicates of each farming system (conventional management, organic management, transition from conventional to organic management, and nature management). Maize yield was obtained by the average of three repetitions measuring 3 m × 3.5 m in five lines for each treatment, excluding the borders. The maize was rainfed and adjusted to 15% moisture content after harvest. Four treatments were evaluated, and they are described below.

The conventional management (**CM**) followed all rules of this management system, except for the use of no-tillage and employed mineral fertilization. We applied 333 kg ha^−1^ of the fertilizer 08-24-12 at sowing and 666 kg ha^−1^ of 20-05-20, 15 days after sowing (DAS). The split application was adopted to minimize N loss by leaching. The total nitrogen input was 160 kg ha^−1^, being ammonium sulfate its principal N source. To control the weeds, we used one application of the herbicides Atrazine and Benzoylcyclohexanedione 15 DAS. To control the caterpillar, we applied (1 L/ha) Connect^®^ (Bayer) at approximately 15 and 22 DAS. The previous history of soil correction consisted of 2 tons ha^−1^ of limestone and 500 kg ha^−1^ of rock phosphate in the year 2015–2016, as well as, 5 tons ha^−1^ of limestone in the year 2015–2016.

The organic management (**OM**) was composed by composted poultry manure. This site has been under organic management for at least five years and was converted from conventional to organic through the same transition management as proposed for one of our treatments above. For the fertilization, a total of 4 tons ha^−1^ of composted poultry manure was added between 10–15 days before sowing (DBS). The total N input was 120 kg ha^−1^ for each crop year. Mechanical and manual weed harrowing were performed to control the weeds. To control the caterpillar, we applied (0.1 L/ha) Tracer^®^ (Spinosad) (Dow AgroScience) approximately 15 DAS. The previous history of soil correction consisted of 2 tons ha^−1^ of limestone and 500 kg ha^−1^ of rock phosphate in the year 2015–2016, as well as, 5 tons ha^−1^ of limestone in the year 2016–2015.

Our third management is composed by a transition field from the conventional cropping to the organic management (**TM**). All the procedures of weed and caterpillar controls followed the CM treatment. For the fertilization in the first year, we applied 2 tons ha^−1^ of composted poultry manure (with a C/N of 13) 10–15 days before sowing (DBS), representing 40% of the total nitrogen fertilization for this year. For the other 60% of fertilization, we applied 200 kg ha^−1^ of fertilizer 04-14-08 at sowing and 400 kg ha^−1^ of fertilizer 20-05-20 approximately 15 days after sowing (DAS). The total nitrogen input in the first year was 148 kg ha^−1^. In the second year, it was 3 tons ha^−1^ of composted poultry manure 10–15 DBS, representing 65% of the total nitrogen fertilization for this year. For the other 35% of fertilization, we applied 200 kg ha^−1^ of fertilizer 04-14-08 at sowing and 200 kg ha^−1^ of 20-05-20 approximately 15 days after sowing (DAS). The total nitrogen input in the first year was 138 kg ha^−1^.

The last management evaluated was nature management (**NM**), *i.e.,* organic management based on green manure, following natural farming principles proposed by the philosopher Mokiti Okada. This site is under the proposed organic management for at least 15 years. The fertilization was composed of castor bean pie (1 ton ha^−1^) and Bokashi (200 kg ha^−1^) was added between 10–15 days before the sowing (DBS). The C/N ratio of castor bean pie and Bokashi are 8 and 15, respectively. Bokashi is a fermented organic fertilizer, which includes the addition of effective microorganisms to improve the process. In this experiment, Bokashi was fermented for three days at 38 °C and was formulated with 39% of wheat bran, 39% of rice bran, 20% of castor bean pie, and 2% of equal parts of a soil mixture collected at 0–10 cm depth. The soil mixture used was composed by a sample from each treatment site, including a forest soil sample. The total N input was 60 kg ha^−1^ for each crop year. Mechanical and manual weed harrowing were performed to control the weeds. To control the caterpillar, we applied (0.1 L/ha) Tracer^®^ (Spinosad) (Dow AgroScience) approximately 15 DAS.

### Soil and plant sampling

The soil and plant sampling procedures occurred during the maize crops 2017–2018 and 2018–2019. Each experiment was composed of 12 plots. Soil samples were obtained during the V5 (vegetative), R1 (reproductive) stage, and seven days after harvest (AH), considering 0–10 cm depth. Each soil sample was composed of a mix of 10 sub-samples randomly selected within each. At the same time, five plants of management were harvested at the stages V5 and R1 for chemical analyses. We sampled a total of 72 soil samples and 48 plants. The sampled soil was sieved through a 3.0 mm diameter sieve, and all living plant material, visible organisms, and stones were removed. The sieved soil samples were freeze-dried at −80 °C. Shoots were weighed, dried at 65 °C for 72 h and ground.

### Soil fertility, soil enzyme activity, and plant nutrient analyses

Soil macronutrients were determined following Van Raij et al. (2001). Dry biomass was used to determine N, P, and K shoot concentrations in plants, according to Malavolta, Vitti & Oliveira (1997). The soil enzyme activity of β-glucosidase (E.C. 3.2.1.21), acid phosphatase (E.C. 3.1.3.2), and alkaline phosphatase (E.C. 3.1.3.1) was determined according to Tabatabai (1994) using 0.5 g of soil. Finally, labile C as permanganate-oxidizable C (POXC) in soils was determined according to Culman et al. (2012) , using 2.5 g of soil.

### High-throughput sequencing of soil community bacteria

Total DNA was extracted using 0.4 g of soil with the PowerSoil DNA Isolation Kit (MoBio Laboratories, Solana Beach, CA, USA), according to manufacturer’s instructions. Bacterial and Archaeal communities were sequenced based on the hypervariable region V4 of 16S rRNA gene using the primers 515F - 806R (Apprill et al., 2015; Parada, Needham & Fuhrman, 2016), and the protocol proposed by earth microbiome (https://earthmicrobiome.org/protocols-and-standards/16s/). All the amplicons were sequenced using Illumina MiSeq V2 (2 × 250 pb) chemistry. The raw sequences were analyzed using the QIIME2 software (Bolyen et al., 2019). Briefly, a divisive amplicon denoising algorithm (DADA2) was used to merge paired reads; the sequences were filtered according to their quality, denoised, the chimeras were removed and it was created a feature table (Callahan et al., 2016) with the plugin *dada2 denoise-paired*. This algorithm uses a model-based approach to correct Illumina amplicon errors, producing a feature table of frequency containing amplicon sequence variants (ASV), which could be considered a putative error-free (representative) sequence for each sample. ASVs were taxonomically assigned to using *feature-classifier classify-sklearn* by SILVA SSU (v132) database. Representative sequences were aligned using MAFFT (Katoh & Standley, 2013) and the phylogenetic tree was produced using FastTree algorithm (Price, Dehal & Arkin, 2010), using the plugin *phylogeny align-to-tree-mafft-fasttree*. The plugin *taxa filter-table* was used to remove Archeal sequences from the dataset, keeping just the bacterial sequences for further analyses. The alpha and beta diversity metrics were calculated using the plugin *diversity core-metrics-phylogenetic*. All samples were rarified at 19,167 sampling depth. Finally, the data were exported for further analyses with *tools export* plugin. The sequence data in this study were deposited into SRA database with the BioProject PRJNA630490.

### Statistical analysis

The boxplot analysis was used to compare the differences of crop yield and plant nutrition values across the treatments. A principal component analysis (PCA) with a biplot was performed to identify the main factors correlated with the soil enzyme activity and soil fertility. The statistical analyses were performed in the R platform using the packages *ggplot2, laercio,* and *FactoMiner*. To evaluate the changes in the α-diversity from the bacterial community, we employed the boxplot analysis. The α-diversity metrics used in this study were Faith’s phylogenetic diversity (faith PD), good coverage, observed ASV richness, and evenness. Distance-based redundancy analysis (db-RDA) was used to evaluate the relationship between soil bacterial community composition (Morisita-Horn dissimilarity distance), soil enzyme activity and soil fertility. DESeq2 analysis was used to calculate the ASV differential abundance among the soil treatments. This analysis fits a generalized linear model with a negative binomial distribution for normalized value for each ASV and to test its differential abundance using a Wald test (Love, Huber & Anders, 2014). The *p*-values were adjusted by multiple testing using the procedure of Benjamini & Hochberg (1995), and selected a false discovery rate (FDR) of 5% to denote statistical significance (Love, Huber & Anders, 2014; Whitman et al., 2016). Finally, the selected ASVs by the differential abundance analysis were correlated with soil enzyme activity and soil fertility data, using a Spearman correlation. All these procedures were performed in the R platform using the packages phyloseq, vegan, DESeq2, coorplot and Hmisc.

## Results

### Yield, plant nutrients, and rainfall

**Figure 1:**
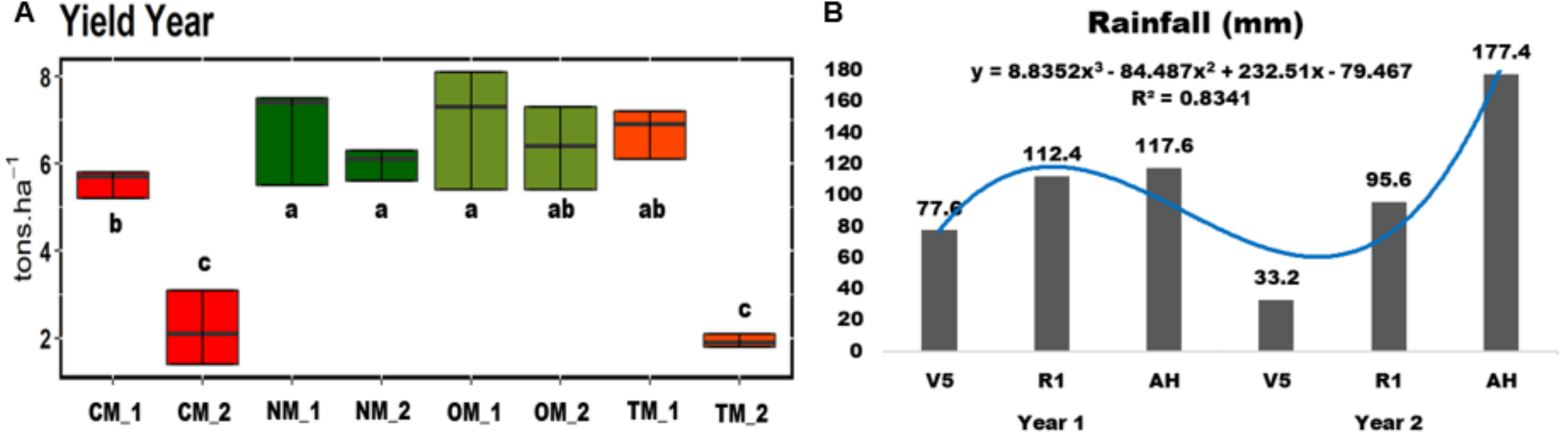
(A) Box-plot of the yield of the two years of the maize experiment and (B) bar plot of the cumulative rainfall 15 days before the soil sampling of the two years of the experiment. (A) The lower and upper box boundaries represent the 25th and 75th percentiles, respectively, the central line stands for the inside box median. CM, conventional management; TM, transition management; OM, organic management; NM, nature management. _1: Year 1, _2: Year 2. (B) The blue line represents a polynomial regression of the third order. V5: V5 vegetative phenological stage, R1: R1 reproductive phenological stage and AH: AH after harvest.

The CM treatment showed the lowest yield among all treatments during the two years (Fig. 1A). For the first year, both organic treatments produced a yield of 7 tons per hectare approximately. There were no differences between these treatments (NM and OM) for the first and the second years. Nonetheless, the treatments with mineral synthetic fertilization (CM and TM) showed a decrease of more than 50% in the yield of the second year. The rainfall that accumulated 15 days before each soil sampling during the two years is shown in Fig. 1B. These results demonstrate a decrease in rainfall values for the second year, especially close to the stages V5 and R1 (Fig. 1B). The nutrient uptake by plants (N, P, and K) showed no differences between all treatments (Fig. S1). Nevertheless, there was a tendency for all three nutrients for a high absorption at the first phase (V5) for CM and TM, whereas the organic treatments absorbed less at this phase, although the absorption at R1 was the opposite for the same treatments (Table S1).

### Changes in the soil chemical attributes and soil enzyme activity

The results from chemical attributes and soil enzyme activity were evaluated by ANOVA (Table 1). For both years, the organic systems (OM and NM) have higher values of Yield, pH, Phosphorus (P), Calcium (Ca), and lower values for Potassium (K) and Aluminum (Al) when compared with the conventional treatment (CM). Also, the transition treatment (TM) has the same results as the organic systems for the variables described above. The enzymatic activity demonstrate high values of alkaline phosphatase and β-glucosidase for NM, and high values of acid phosphatase in the second year of the experiment for CM.

**Table 1:** Mean of the soil chemical attributes and enzymes. Letters were attributed after Tukeys test. Uppercase letters are the differences between the treatments for the same year. Lowercase letters are between years 1 and 2 for each treatment. Values in bold showing significant variation, considering p-value <0.05 (ANOVA). POXC: labile carbon, N.inorg: inorganic nitrogen (NH4+ + NO3-), C/N inorg: the ratio between POXC and N.inorg. CM: conventional management, TM: transition management, OM: organic management, NM: nature management.

	CM		NM		OM		TM	
Year	1	2	1	2	1	2	1	2
Ammonium	11.15 Aa	16.17 Aa	8.60 Aa	8.29 Aa	8.93 Aa	8.35 Aa	9.18 Aa	10.69 Aa
Nitrate (NO_3_^−^)	14.60 Aa	22.09 Aa	***6.85 Ab***	***12.85 Aa***	***6.61 Ab***	***13.53 Aa***	12.91 Aa	18.19 Aa
N inorg	25.75 Aa	38.26 Aa	15.45 Aa	21.14 Aa	15.54 Aa	21.89 Aa	22.08 Aa	28.88 Aa
POXC	421.40 Aa	454.00 Aa	475.79 Aa	506.62 Aa	515.88 Aa	472.11 Aa	546.10 Aa	499.01 Aa
C/N inorg	24.69 Aa	18.73 Aa	31.81 Aa	28.16 Aa	35.30 Aa	27.10 Aa	34.12 Aa	24.28 Aa
β-glucosidase	***35.00 Bb***	***66.59 ABa***	***65.73 Ab***	***81.03 Aa***	***37.30 Bb***	***63.36 ABa***	***32.38 Bb***	***57.56 Ba***
Acid Phosphatase	***292.33 ABb***	***494.90 Aa***	***301.51 Ab***	***417.94 Ba***	***284.49 Ab***	***412.97 Ba***	***247.98 Bb***	***388.08 Ba***
Alkaline Phosphatase	***61.01 Bb***	***91.37 Ba***	***146.61 Aa***	***168.64 Aa***	***67.67 Bb***	***113.32 Ba***	***64.87 Bb***	***104.69 Ba***
pH	***4.72 Ca***	***4.71 Ca***	***5.73 Aa***	***5.82 Aa***	***5.69 Ab***	***5.90 Aa***	***5.37 Bb***	***5.57 Ba***
Organic matter	27.78 Aa	28.00 Aa	26.33 Aa	26.11 Aa	27.00 Aa	25.22 Aa	27.56 Aa	26.22 Aa
Phosphorus (P)	***7.89 Bb***	***9.44 Ba***	***59.22 Aa***	***40.78 Aa***	***17.89 ABb***	***30.44 Aa***	***16.67 ABb***	***36.22 Aa***
Potassium (K^+^)	***6.91 Ab***	***9.18 Aa***	***4.54 Ba***	***4.64 Ca***	***4.08 Ba***	***5.27 Ca***	***6.51 Aa***	***7.47 Ba***
Calcium (Ca^+2^)	***34.11 Ba***	***28.67 Ba***	***54.56 Aa***	***54.22 Aa***	***48.67 Aa***	***51.33 Aa***	***51.89 Aa***	***59.89 Aa***
Magnesium (Mg^+2^)	***15.11 Ba***	***13.67 Ca***	***24.00 Aa***	***25.67 Aa***	***17.78 Ba***	***16.44 BCa***	***18.22 Ba***	***19.33 Ba***
Aluminium (Al^+3^)	***3.56 Aa***	***2.78 Aa***	***0.02 Ba***	***0.02 Ba***	***0.02 Ba***	***0.02 Ba***	***0.13 Ba***	***0.02 Ba***
Yield	***5.57 Ba***	***2.20 Bb***	***6.80 Aa***	***6.00 Ab***	***6.93 Aa***	***6.37 Aa***	***6.73 Aa***	***1.93 Bb***

**Figure 2:**
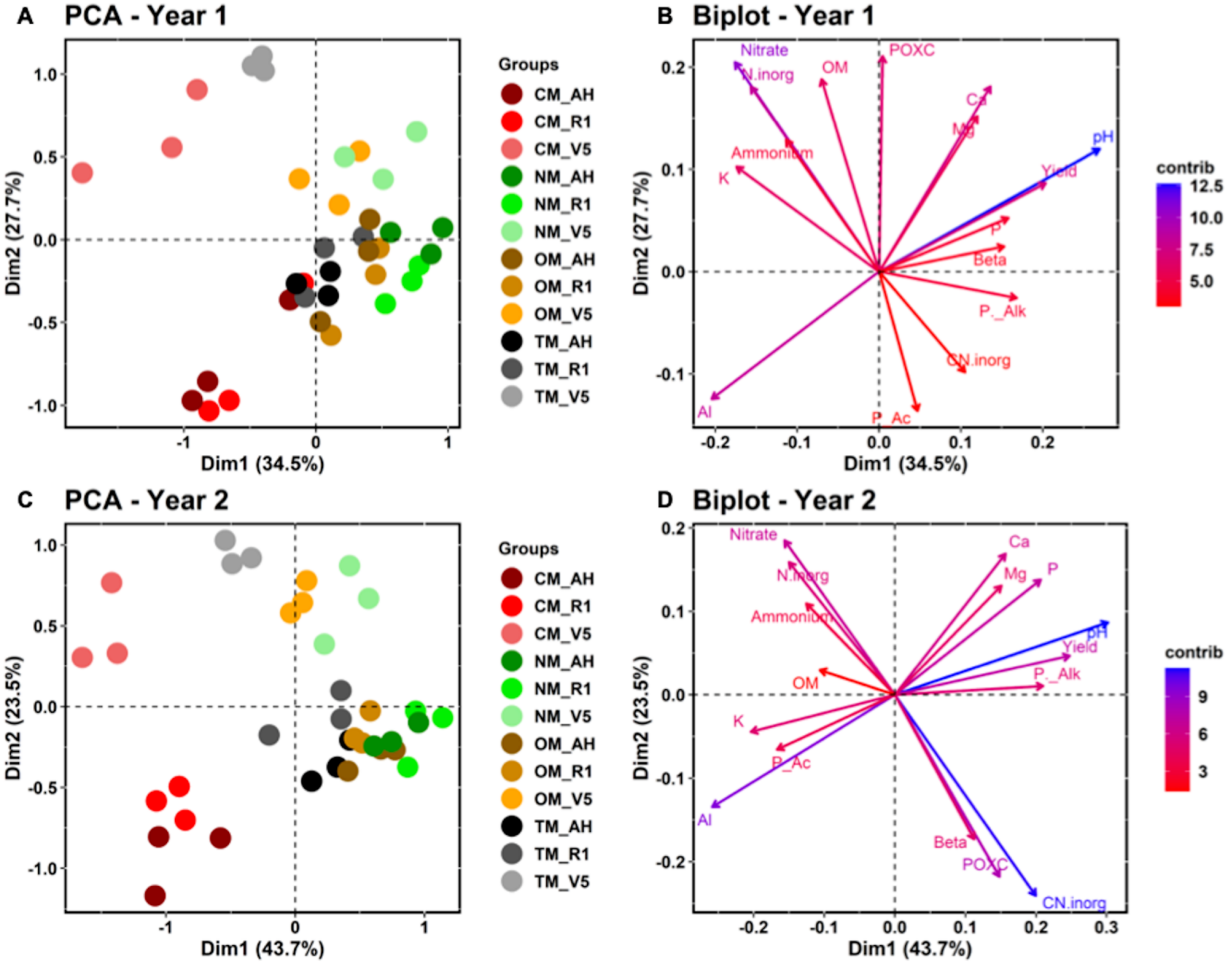
Principal component analyses (PCA) divided into treatments (A and C) and variables (B and D), such as the soil chemical attributes and soil enzyme activities measured during the experiment. The graphics (A and B) and (C and D) represent the samples from the first and second years of the experiment, respectively. P_Ac, Acid phosphatase; P_Alk, Alkaline phosphatase, Beta, β-glucosidase activity, POXC: labile carbon, OM, organic matter, N.inorg, inorganic nitrogen (NH4+ + NO3-); CN_inorg, the ratio between POXC and N.inorg. In the legend V5, sample from V5 stage; R1, samples from R1 stage, and AH, samples from after harvesting stage. CM, conventional management; TM, transition management; OM, organic management; NM, nature management.

The principal components analysis (PCA) was used to integrate all the variables of the soil chemical attributes and soil enzyme activity and establish their correlations between samples and attributes. The results show that the sum of the first and second components of the PCA explained 62.2 and 67.2% for the datasets in the first and second years, respectively (Fig. 2). For both years, two PCA agroupments can be observed. The first is composed by the samples from CM treatment. The second group was composed of all the other samples, including the organic systems and the transition treatment (Figs. 2A and 2C). According to the biplot from our PCA, we found that Yield, pH, P, alkaline phosphatase, and β-glucosidase are positively correlated with the second group (Figs. 2B and 2D). The Al and K attributes correlated positiviley with the first group, followed by the nitrate which correlated positively with the samples CM_V5 and TM_V5 due to the proximity of these samples retrieved with the second mineral fertilization (15 DAS).

### Changes in the bacterial community

**Figure 3:**
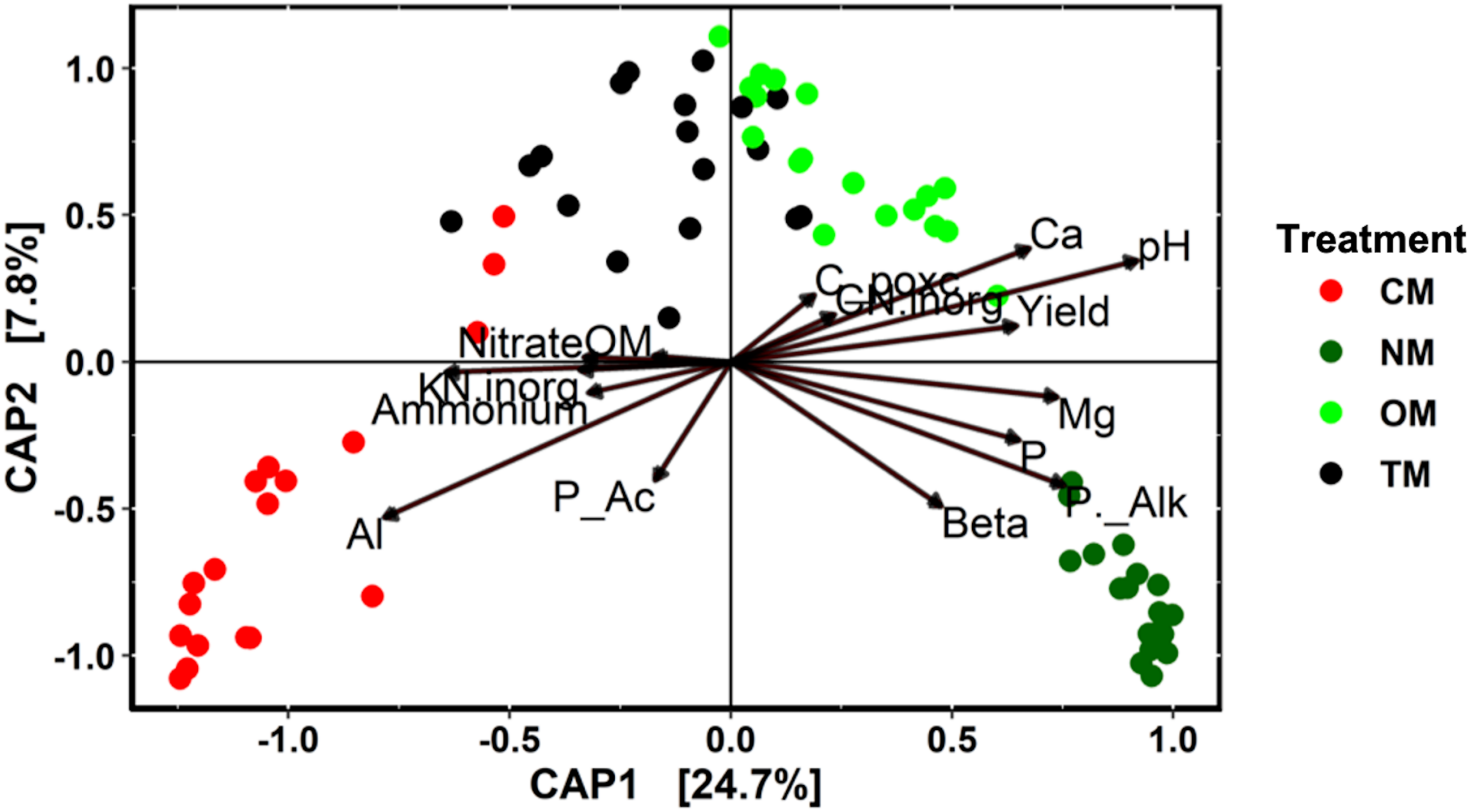
Distance-based redundancy analysis (db-RDA) based on the Morisita-Horn dissimilarity index, considering the sequence data of the soil chemical attributes, and soil enzyme activity as an environmental matrix. P_Ac, Acid phosphatase; P_Alk, Alkaline phosphatase; Beta, β-glucosidase activity; C_poxc, labile carbon; OM, organic matter, N. inorg, inorganic nitrogen (NH4+ + NO3-); CN_inorg, the ratio between C_poxc and N.inorg. CM, conventional management; TM, transition management; OM, organic management; NM, nature management.

**Figure 4:**
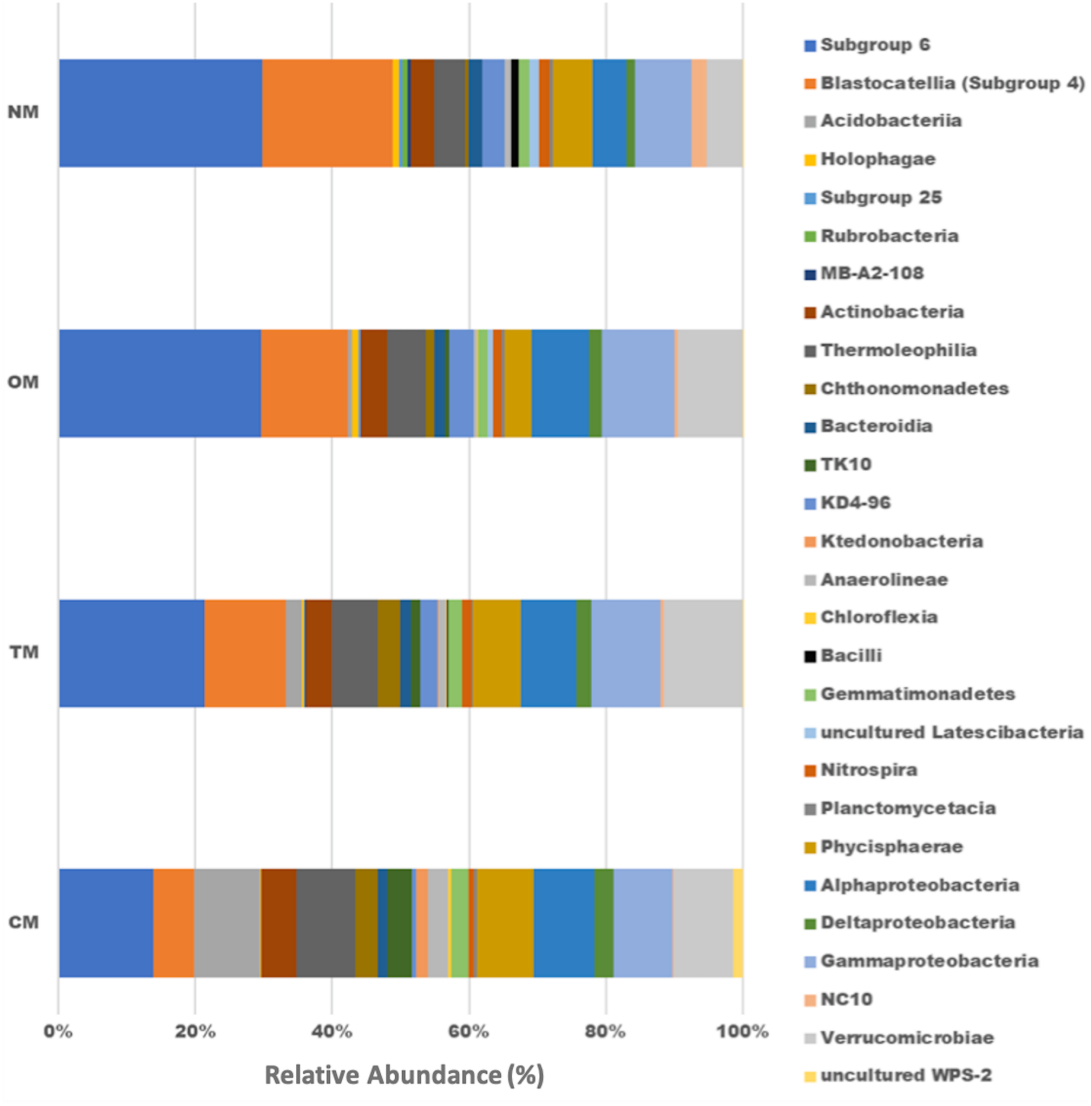
Bar plot of the abundance for bacterial classes. The abundance table was built with the 481 ASVs selected by DEseq2. CM, conventional management; TM, transition management; OM, organic management; NM, nature management.

**Figure 5:**
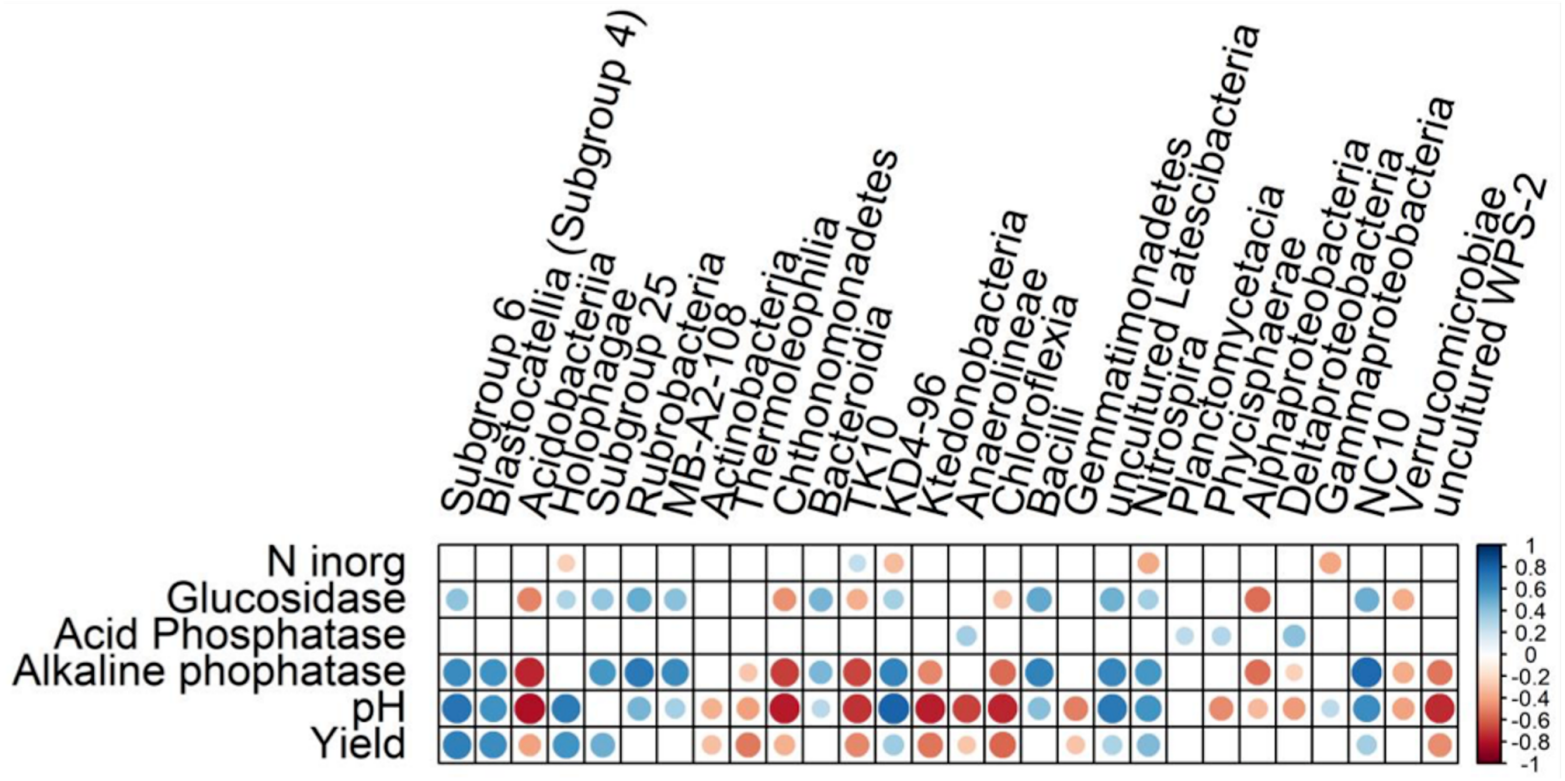
Spearman’s rank correlation between bacterial classes and environmental variables. The red and blue dots represent the negative and positive Spearman’s rank correlation coefficient (r), considering *p* value < 0.05, respectively. The classes were selected after the Deseq2 analysis and the environmental variables after the db-RDA permutation test.

The diversity and richness indices had non-significant differences among the experimental treatments evaluated in this study (Fig. S2). Comparing these indices between the cropping years, in the second year (2018–2019), we obtained higher faith PD diversity and richness or observed ASV values (Fig. S2). In Fig. 3, the db-RDA shows a clear distinction of the bacterial community structure among the treatments, where the soil samples tend to group according to the source of fertilization (mineral, composted poultry manure, or green manure). It was observed that the same chemical attributes and enzyme activity were the principal drivers of bacterial community structure in both years (Table 2). The treatments OM and TM used composted poultry manure and grouped together, without a clear correlation with any soil attribute; while the CM and NM showed the highest differences across the treatments. We observed that the bacterial community in CM correlated with inorganic N, ammonium, nitrate, Al, and K, while in NM they correlated clearly with pH, alkaline phosphatase, β-glucosidase, Mg, labile carbon, and P (Fig. 3). We established significant correlations between the ASVs and each soil management (Deseq2 analysis), which permits identifying the most abundant classes associated with soil management. Deseq2 analysis resulted in 481 ASVs and a total of 420,448 sequences at 5% of significance. The classes Subgroup 6 and *Blastocatellia* from the phylum Acidobacteria, and Nitrospira from the phylum Nitrospirae were more abundant in the treatments that received organic fertilization. For NM, the class *Bacilli* (phylum Firmicutes), and NC10 (Rokubacteria) had an increase in their abundance when compared to the CM treatment. We also observed that the CM treatment had a higher abundance in classes like Verrucomicrobiaceae (phylum Verrucomicrobia), TK-10, Chloroflexia, and Anaerolineae from the phylum Chloroflexi; Phycisphaerae (phylum Planctomycetes), Acidobacteriia (phylum Acidobacteria); and Thermoleophilia (phylum Actinobacteria) (Fig. 4). The Spearman correlation showed positive and negative results with Yield, pH and alkaline phosphatase for the classes previously described as more abundant in treatments NM and CM, respectively (Fig. 5). The β-glucosidase activity correlated with the previous results described, and class *Blastocatellia* was the exception with no significant correlation.

**Table 2:** Permutation model of the distance-based redundancy analysis (db-RDA), considering a forward-selected environmental variable stepwise search.

Attributes	F	Pr(>F)	
pH	11.3932	0.001	*
Alkaline phosphatase	9.3514	0.001	*
β-glucosidase	7.6794	0.001	*
N.inorg	5.5795	0.001	*
Ammonium (NH_4_^+^)	4.3798	0.001	*
Nitrate (NO_3_^−^)	2.7699	0.004	*
Magnesium (Mg^+2^)	2.6721	0.008	*
Aluminum (Al^+3^)	2.4734	0.011	*
POXC	2.3991	0.007	*
Potassium (K^+^)	1.842	0.039	*
Phosphorus (P)	1.7746	0.042	*
Acid phosphatase	1.5661	0.101	
CN.inorg	1.4846	0.109	
Calcium (Ca^+2^)	1.4161	0.109	
Yield	1.291	0.159	
Organic matter	1.2715	0.172	

**Notes.**

N.inorg inorganic nitrogen (NH^4+^+ NO^3-^) * Significant values, considering *p*-values <0.05

## Discussion

Organic farming appears as an alternative practice that avoids environmental problems and the impacts on ecosystem services. Besides the benefits of this environmentally friendly management, several studies have suggested the low yield averages as its main problem (Seufert, Ramankutty & Foley, 2012). Also, the absence of scientific information contributes to maintain this low yield scenario limiting the development of organic practices. Here, we evaluated the soil bacterial community across different organic practices (OM and NM) and conventional management (CM). The Mokiti Okada Research Center presents an internal practice suggesting that the adoption of organic management requires five years of a slow transition from one management system to the other to recuperate the natural fertility of the soil. For this reason, we included the transition management (TM) in our experiment. Previous maize experiments have compared conventional and organic managements used more than 160 kg ha^−1^ of N in the form of green and poultry manures (Fernandez et al., 2016; Wang et al., 2017). In our experiment, the organic systems with low N-input (120 kg ha^−1^ as composted poultry manure for OM, and 60 kg ha^−1^ of composted green manure plus 60 kg ha^−1^ of Bokashi for NM) resulted in yield enhancement and changes in the bacterial community structure, which may improve the soil nutrient cycling, especially for carbon and phosphorus (as will be discussed later).

In the first and second years, the crop yields for the two organic systems (OM and NM) were higher than conventional management (CM), which is based on mineral fertilization. This result agrees with the Yield average of 6.2 and 5.5 ton/ha at São Paulo State in Brazil during the harvests 2017/2018 and 2018/2019 (CONAB, 2018; CONAB, 2019). During the second year, we observed a reduction in rainfall during the stages V5 and R1, which might have negatively affected the yield for the treatments that received the synthetic fertilizers (TM and CM). Interestingly, in the organic treatments (OM and NM) there was no loss of yield detected. Data of rainfall and nutrient absorption (Fig. 1 and Table S1) compared at different phenological phases showed an initial gain of nutrients that was not followed by high growth (CM and TM), suggesting a water deficit just before R1. This period normally measures the most intense growth phase as shown by the organic treatments (NM and OM), which did maintain a higher water stock in the soil because of their high contents of organic matter and, therefore, maintained a high growth rhythm. High productivity for organic farming under severe drought conditions has been observed in previous studies (Lockeretz, Shearer & Kohl, 1981; Lotter, Seidel & Liebhardt, 2003). This gain was associated with the potential of soil organic matter to retain moisture and improve soil aeration. In general, our results had lower values of Yield when compared with these studies, which was associated with a drought period. However, we would like to emphasided the severe impact of the drought condition on conventional maize production compared with the organic farming practices. Future studies should be necessary to understand the changes in soil attributes and the role of the microbial community that are correlated with drought resistance. For the plant nutrition, we did not observe differences between nutrient uptake. Thus, our results demonstrate that composted poultry manure (OM) and green manure + Bokashi (NM) can supply the nutrient requirements for maize growth. After composting, the poultry manure is quickly mineralized in soil because of its low C/N ratio, increasing nutrient availability (Berry et al., 2002; Sistani, Sikora & Rasnake, 2008). Pereira et al. (2018) also demonstrated that composted organic materials generally present a greater nutrient content (primarily for carbon and phosphorus) than fresh organic matter and are much more indicated to be used in organic treatments.

Notwithstanding, our survey demonstrated that using green manure + Bokashi may present complementary benefits because Bokashi acts as an immediately available probiotic food for the microbiota. Green manure usually has a high C/N ratio when not composted, which results in N immobilization and a decrease of crop yields in the first years (Baggs, Watson & Rees, 2000). However, the fermentation process of the green manure in the Bokashi production may increase the microbial activity and provide a better nutrient uptake by the plants. Bokashi was developed in Japan and is widely applied to increase soil organic matter and crop growth improvement in the root system. The lactic acid in Bokashi plays a role in the green manure decomposition, besides fortifying the microbiota. Previous studies have demonstrated the efficiency of Bokashi to increase yields in sweet potato and maize (Dou, Komatsuzaki & Nakagawa, 2012; Yuliana, Sumarni & Islami, 2015).

A meta-analysis recently showed that organic farming enhances the carbon and the nitrogen of microbial biomass, total phospholipid fatty-acids, dehydrogenase, urease, and protease activities (Lori et al., 2017). Our results showed that soil pH, P, and the soil enzyme activity, such as the phosphatase and β-glucosidase, are the most responsible variables to explain the differences between the organic and mineral synthetic fertilization. Although most of these results stem from the temperate climatic zone, our results suggest the same interpretation in the tropical zone. Mineral fertilization like ammonium and phosphorus impact the soil microbial properties by liberating H^+^ and decreasing soil pH (Bünemann, Schwenke & Van Zwieten, 2006), or contaminating the environment by liberating heavy metals (McLaughlin et al., 2000). In contrast, organic material enhances the soil buffering capacity and chelates Al^3+^, resulting in soil pH increases (Whalen et al., 2000; Baolan et al., 2014). Many soil enzymes have their activities in soil increased when pH values increase. Previous studies have demonstrated that β-glucosidase and alkaline phosphatase are positively correlated with soil pH (Eivazi & Tabatabai, 1990; Dick, Cheng & Wang, 2000), corroborating our results.

The increased activity of alkaline phosphatase with organic fertilization correlated positively with the P content in the soil, especially for the NM treatment. Phosphorus contents in soils are usually unavailable for plants for multiple reasons, including the immobile forms of P in soil (Halvorson & Black, 1985), the fixation in acid tropical soils, and sometimes even because of leaching (Borda et al., 2011). It has been observed that the addition of organic fertilizers reduces soil P fixation, permitting P uptake by the plants (Malik, Marschner & Khan, 2012). Our results suggest that an enhance in alkaline phosphatase activity may improve the availability of P even in tropical soils where acid conditions of the soil are common. We observed that the addition of green manure resulted in the highest value for β-glucosidase activity. β-Glucosidase catalyzes the hydrolysis in the final step of cellulose decomposition producing glucose and is considered an important soil indicator for the degradation of green manure inputs in soil (Turner et al., 2002). Many bacteria groups in soil are induced to synthesize great amounts of β-glucosidase, which is excreted into the environment where it acts in the catalysis of cellobiose. After β-glucosidase breaking cellobiose into two glucose molecules, these are assimilated by the bacteria because the cells present sugar transporters in their cell membranes. It is important to emphasize that no differences were observed in ammonium or nitrate contents in soil, even for the low N input treatments in the organic treatments. The P availability has been reported to correlate with biological nitrogen fixation because of the high necessity of phosphorus to supply the energetic demand for ATP during this process (Zheng et al., 2016; Rousk et al., 2017). Thus, nitrogen fixation affects both the available N and P contents. N and P are driven by β-glucosidase and alkaline phosphatase, respectively, so that both enzymes were favored by the organic managements.

We used the high-throughput sequencing of the 16S rRNA gene to evaluate the α- and β-diversity shifts, and identify the specific bacterial groups associated with soil biochemistry across the organic and conventional farming systems. Non-significant changes in α-diversity indices were observed between the treatments evaluated. Different effects of the organic and conventional management on the soil microbial community have been suggested, which may increase, decrease or maintain the microbial diversity. (Hartmann et al., 2015; Lupatini et al., 2017; Liu et al., 2007; Reilly et al., 2013). Studies that reported an increase in the microbial diversity indices after organic fertilization also demonstrated an increase in C contents in soil (Hartmann et al., 2015). In our study, we did not observe high increases in C and N contents of the soil, which may explain the similar diversity indices observed between the treatments.

The distance-based redundancy analysis allowed us to observe the changes in the β-diversity of the bacterial community in the organic and conventional managements, identifying the key soil parameters related to the variance. These results show a high correlation of the soil microbiome with enzyme activity and soil fertility, suggesting that a high turnover in soil microbial communities is an early predictor of soil organic matter cycling. In our study, this statement also occurred in TM, where the bacterial community structure is more similar to OM than to CM, even though the transition treatment received only 40% of the total fertilization with organic sources. Previous studies reported that soil pH is one of the major drivers of microbial community composition in pristine and agronomic environments (Fierer & Jackson, 2006; Griffiths et al., 2011). Soil chemical properties and enzyme activity are also highly correlated with changes in the microbial community (Zhang et al., 2019; Chen et al., 2020). Our results demonstrated that the most important bacterial groups that correlate positively with CM are negatively correlated with the soil pH, alkaline phosphatase and β-glucosidase activities.

The most abundant bacterial phyla for the CM treatment are Verrucomicrobia, Chloroflexi, Planctomycetes, and Actinobacteria. Organic farming (NM and OM) contains Acidobacteria (classes Subgroup 6 and Blastocatellia), Firmicutes (class Bacillus), Nitrospirae and Rokubacteria as the most abundant phyla. Previous studies that evaluated bacterial community changes in temperate climate reported an abundance increase of *Acidobacteria* and *Planctomycetes* in organic systems (Lupatini et al., 2017), while the phyla *Actinobacteria* and *Chloroflexi* are associated with conventional farming (Li et al., 2012). Our results in a tropical climate confirmed that these groups are highly present in organic and conventional systems, except for the phylum *Planctomycetes*, and also demonstrated additional phyla correlated with these agronomic practices.

Contrasting results for *Actinobacteria* and *Acidobacteria* are common in the literature when these groups are compared with soil pH. Previous studies have demonstrated that *Actinobacteria* have a strong positive correlation with soil pH (Lauber et al., 2009), but this effect was not confirmed in conventional arable soils (Rousk et al., 2010). Here, the class *Thermoleophila* (phylum *Actinobacteria*) had an apparent abundance decrease in the organic fertilization and showed a negative correlation with soil pH and alkaline phosphatase. *Acidobacteria* has been described as an oligotrophic group that prefers acid soils and the addition of organic fertilizers in soils is expected to inhibit its growth (Fierer & Jackson, 2006). Rousk et al. (2010), however, demonstrated that class *Subgroup 6* (phylum *Acidobacteria*) has a positive correlation with soil pH. Recently in tundra soils, the authors showed distinct habitats preferences of *Blastocatellia* and *Acidobacteriia, being the first* positively correlated with soil pH and negatively correlated with nitrogen availability, while the second has the opposite tendency (Ivanova et al., 2020). Our results also demonstrated a positive correlation with soil pH for *Subgroup 6*, and *Blastocatellia* and a negative correlation for class *Acidobacteriia*, confirming that some phylogenetic affiliations may have distinct ecological characteristics in agronomic soils. Besides, these contrasting results in the correlation with soil pH for *Acidobacteria* and *Actinobacteria* may have been related to additional characteristics of the soil (*e.g.*, soil enzymes, C and P availability) or management practices (*e.g.*, organic fertilization). Former studies have reported phyla like *Acidobacteria*, *Actinobacteria*, *Chloroflexi*, *Cyanobacteria*, *Firmicutes*, *Gemmatimonadetes*, *Planctomycetes*, *Proteobacteria,* and *Verrucomicrobia* are the most abundant ones, able to produce alkaline phosphatase in soil (Fernandez et al., 2016; Ragot et al., 2010). In our results, most of these groups are present as significant phyla in conventional or organic systems, but only the phyla *Acidobacteria* and *Firmicutes* (both associated with the organic systems) were positively correlated with alkaline phosphatase. Thus, the adoption of organic practices increased specific phyla associated with alkaline phosphatase activity, which permits better P cycling in this environment. The phyla *Rokubacteria* and *Nitrospira* associated with organic systems, especially NM, may be considered crucial for C cycling and N nitrification. The first phylum has been recently described in Amazon soils, showing the capacity to metabolize complex hydrocarbonates (Kroeger Marie et al., 2018). Here, our results demonstrate that this phylum is positively correlated with β-glucosidase activity in soil, which suggests that *Rokubacteria* is involved in green manure degradation. *Nitrospira* has been described to perform the nitrification process in soil (Daims et al., 2015), and its higher abundance in organic systems suggests that this group was promoted by the low input of N in these treatments.

Our study confirmed the hypothesis that organic farming may promote superior productivity of maize when compared with conventional farming. A possible reason for the varying results of the yield production in organic management may be associated with the quality of soils used in the experiments. Most experiments comparing organic and conventional management systems are initialized on a soil that so far had been cultivated in a conventional system, using a soil that had been partially degraded and that only produces high yields when fertilized with great amounts of synthetic fertilizers and many other chemical inputs. For this reason, we included a transitional system of five years before calling the system organic. Previously to implanting an organic system at a site that had been cultivated using a conventional system, the soil has been recovered obtaining all biological characteristics of a healthy soil that are the base for a successful organic culture system. Otherwise, it is probable to get a failure. Thus, further studies should consider the conditions of the abiotic and biotic factors before the implantation of an organic management system. Based on our results, we suggest that making the correct transitional period from conventional to organic management improves a healthy and quite stabilized fertile soil, improved in organic matter cycling and microbiota which sustains plant nutrition and development. Finally, we confirmed that the organic managements, including the natural and the so-called organic management, promotes changes in the soil bacterial community and its functionalities.

## Conclusions

Our data demonstrate that the organic farming systems, both with low N input and scientifically selected specific organic fertilization, could compete with the crop yield of conventional farming or even surpass it. Soil improvements observed in the organic systems correlate with changes in specific groups of the bacterial soil microbiome. Hence, this study contributes to identify beneficial traits of bacteria associated with organic cropping in tropical soils. We suggest future studies use a similar approach in distinct tropical regions to permit manipulation of the soil microbial functions.

## Supplemental Information

10.7717/peerj.11985/supp-1 Supplemental Information 1 Boxplot of the nutrient uptake per plant The lower and upper box boundaries represent the 25th and 75th percentiles, respectively, the central line stands for the inside box median. CM: conventional management, TM: transition management, OM: organic management, NM: nature management.

10.7717/peerj.11985/supp-2 Supplemental Information 2 Boxplot of the diversity indices The lower and upper box boundaries represent the 25th and 75th percentiles, respectively, the central line stands for the inside box median. CM: conventional management, TM: transition management, OM: organic management, NM: natural management. _1: Year 1, _2: Year 2. PD is the Faith’s phylogenetic diversity and Richness is the observed ASVs numbers. Red dots representing outliers.

10.7717/peerj.11985/supp-3 Supplemental Information 3 Median and the standard deviation of the nutrient uptake per plant

10.7717/peerj.11985/supp-4 Supplemental Information 4 Raw data from the experiment

10.7717/peerj.11985/supp-5 Supplemental Information 5 Raw data for cumulative rainfall

## Additional Information and Declarations

### Data Availability

The following information was supplied regarding data availability: The raw data are available in the Supplemental Files. Additionally, sequences are available at NCBI BioProject: PRJNA630490.
