# Supplementary material for: Organic farming practices change the soil bacteria community, improving soil quality and maize crop yields
Source: PeerJ. 2021 Sep 23;9:e11985. doi: 10.7717/peerj.11985 (PMC8465994; doi:10.7717/peerj.11985)
Supplement: Supplemental Information 2 — The lower and upper box boundaries represent the 25th and 75th percentiles, respectively, the central line stands for the inside box median. CM: conventional management, TM: transition management, OM: organic management, NM: natural management. _1: Year 1, _2: Year 2. PD is the Faith’s phylogenetic diversity and Richness is the observed ASVs numbers. Red dots representing outliers. [file peerj-09-11985-s002.pdf]

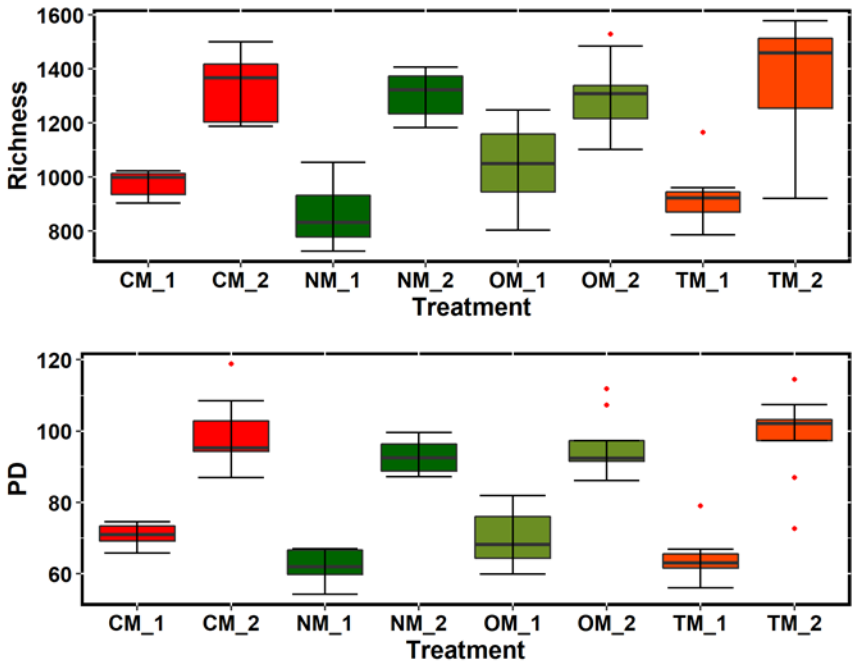

Supplementary Figure 2 Boxplot of the diversity indices. The lower and upper box boundaries represent the 25th and 75th percentiles, respectively, the central line stands for the inside box median. CM: conventional management, TM: transition management, OM: organic management, NM: natural management. _1: Year 1, _2: Year 2. PD is the Faith´s phylogenetic diversity and Richness is the observed ASVs numbers. Red dots representing outliers.
