# Supplementary material for: Organic farming practices change the soil bacteria community, improving soil quality and maize crop yields
Source: PeerJ. 2021 Sep 23;9:e11985. doi: 10.7717/peerj.11985 (PMC8465994; doi:10.7717/peerj.11985)
Supplement: Supplemental Information 3 [file peerj-09-11985-s003.docx]

Supplementary Table 1 – Median and the standard deviation of the nutrient uptake per plant.

| Treatments | Nitrogen (mg plant ^-1^) | Phosphorus (mg plant ^-1^) | Potassium (mg plant ^-1^) |
| --- | --- | --- | --- |
| CM_V5 | 641 ± 150 | 59 ± 15 | 549 ± 86 |
| CM_R1 | 3893 ± 558 | 434 ± 59 | 2379 ± 806 |
| NM_V5 | 412 ± 41 | 31 ± 4 | 300 ± 33 |
| NM_R1 | 3518 ± 593 | 474 ± 82 | 2809 ± 587 |
| OM_V5 | 400 ± 65 | 33 ± 5 | 352 ± 45 |
| OM_R1 | 4705 ± 1089 | 573 ± 64 | 4294 ± 796 |
| TM_V5 | 589 ± 136 | 48 ± 10 | 474 ± 38 |
| TM_R1 | 5389 ± 1128 | 542 ± 74 | 4649 ± 1.508 |
